# Young bone marrow Sca‐1 cells protect aged retina from ischaemia‐reperfusion injury through activation of FGF2

**DOI:** 10.1111/jcmm.13905

**Published:** 2018-09-25

**Authors:** Zhengbo Shao, Jie Wu, Guoqing Du, Huifang Song, Shu‐Hong Li, Sheng He, Jiao Li, Jun Wu, Richard D. Weisel, Huiping Yuan, Ren‐Ke Li

**Affiliations:** ^1^ Department of Ophthalmology The Second Affiliated Hospital of Harbin Medical University Harbin China; ^2^ Research Institute Second Affiliated Hospital of Harbin Medical University Harbin China; ^3^ Laboratory of Medical Genetics Harbin Medical University Harbin China; ^4^ Division of Cardiovascular Surgery Toronto General Hospital Research Institute University Health Network Toronto ON Canada; ^5^ Shanxi Medical University Taiyuan China; ^6^ Department of Cardiology Second Affiliated Hospital of Guangzhou Medical University Guangzhou China; ^7^ Division of Cardiac Surgery Department of Surgery University of Toronto Toronto ON Canada

**Keywords:** aging, retinal ischaemia‐reperfusion, retinal regeneration, stem cell homing

## Abstract

Retinal ganglion cell apoptosis and optic nerve degeneration are prevalent in aged patients, which may be related to the decrease in bone marrow (BM) stem cell number/function because of the possible cross‐talk between the two organs. This pathological process is accelerated by retinal ischaemia‐reperfusion (I/R) injury. This study investigated whether young BM stem cells can regenerate and repair the aged retina after acute I/R injury. Young BM stem cell antigen 1 positive (Sca‐1^+^) or Sca‐1^−^ cells were transplanted into lethally irradiated aged recipient mice to generate Sca‐1^+^ and Sca‐1^−^ chimaeras, respectively. The animals were housed for 3 months to allow the young Sca‐1 cells to repopulate in the BM of aged mice. Retinal I/R was then induced by elevation of intraocular pressure. Better preservation of visual function was found in Sca‐1^+^ than Sca‐1^−^ chimaeras 7 days after injury. More Sca‐1^+^ cells homed to the retina than Sca‐1^−^ cells and more cells differentiated into glial and microglial cells in the Sca‐1^+^ chimaeras. After injury, Sca‐1^+^ cells in the retina reduced host cellular apoptosis, which was associated with higher expression of fibroblast growth factor 2 (FGF2) in the Sca‐1^+^ chimaeras. Young Sca‐1^+^ cells repopulated the stem cells in the aged retina and diminished cellular apoptosis after acute I/R injury through FGF2 and Akt signalling pathways.

## 1 INTRODUCTION

Aging diminishes the functional capacity of stem cells and reduces the ability of the aged organism to repair tissue following injury.^1^ Glaucoma, a sight‐threatening disorder associated with death of retinal ganglion cells (RGCs) and degeneration of optic nerve fibres, affects millions of people worldwide and advanced age is widely recognised as one of the major risk factors for many of the leading causes of vision loss, including glaucoma.^2^, ^3^ The incidence of glaucoma increases exponentially with age.^4^, ^5^ Retinal ischaemia‐reperfusion (I/R) is a pathophysiological process contributing to the cellular damage in glaucoma. Damage to any type of retinal neuron initiates the loss of vision and therapeutic modalities that can reverse these degenerative processes are required to prevent or reverse vision loss. Moreover, aged and diseased tissues provide an unfavourable microenvironment for regeneration.^6^, ^7^, ^8^ Therefore, an intervention to rejuvenate the aged retina may be an important and innovative treatment strategy.^1^, ^9^

Recently, stem cell therapy for degenerative retinal diseases has provided hope to restore vision for otherwise incurable disease processes. Bone marrow (BM) is a potential accessible source of autologous stem cells for retinal cell regeneration without the disadvantage of the immune barrier inherent to allogeneic cell transplants.^10^ The in vivo mobilisation of BM‐derived stem cells (BMSC) can contribute to retinal repair and this regenerative therapy can be implemented without invasive procedures.^11^, ^12^, ^13^ BMSCs have been reported to express a variety of cytokines and growth factors, which have powerful trophic and protective functions for neural tissue‐derived cells. However, the number of these cells is relatively low and complete recovery of the damaged tissues remains elusive, especially in older patients who have limited function of endogenous stem and progenitor cells.^14^

Recently, we evaluated the effect of cardiac‐resident BMSCs on the recovery of cardiac function after injury in aged animals.^15^ We found that BM reconstitution in old recipient mice with young donor cells improved cardiac functional recovery following injury. Furthermore, we demonstrated that the young BM cells from the reconstituted BM stably integrated in the aged myocardium prior to injury and were required for functional recovery after injury.^15^ Subsequently, we identified the stem cell antigen 1 (Sca‐1^+^) cell as the young BM cell type which had the greatest ability to home to the myocardium and protect the aged recipient mouse heart.^16^ Sca‐1 has been widely used as a marker to isolate haematopoietic stem cells. This subpopulation of BM cells has been demonstrated to promote neuronal fibre growth in vitro, and cells expressing early ocular markers were highly enriched within the murine Sca‐1^+^ cells.^17^

Here, we investigated the importance of young BM‐derived Sca‐1^+^ cells for retinal homoeostasis and for retinal repair and regeneration after injury in aged mice. We reconstituted old mice with BM‐derived Sca‐1^+^ or Sca‐1^−^ cells from young green fluorescence protein (GFP^+^) mice to generate Sca‐1^+^ [Y(Sca1^+^)‐O] and Sca‐1^−^ [Y(Sca1^−^)‐O] chimaeras, respectively. Chimaeric mice received retinal I/R to induce injury to the optic nerve. We found that BM chimaerism established with young Sca‐1^+^ cells was associated with better restoration of retina progenitors and improved healing of the aged retina after injury. The improved regeneration of aged retina involved activation of the fibroblast growth factor 2 (FGF2)‐Akt pathway. Suppressing the activation of this pathway using an FGF2 neutralising antibody blocked the beneficial effects of Sca‐1^+^ cells on retinal repair.

## 2 MATERIALS AND METHODS

A detailed methodology can be found in the Supplemental Material.

### 2.1 Animal and BM reconstitution procedures

The Animal Care Committee of the University Health Network approved all experimental procedures, which were carried out according to the Guide for the Care and Use of Laboratory Animals (NIH, 8th Edition, 2011). Young nucleated BM cells were harvested from femurs and tibias of C57BL/6‐Tg‐GFP or C57BL/6 wild‐type mice (2‐3 months, the Jackson Laboratory, Bar Harbor, ME, USA), and separated into Sca‐1 positive‐ and negative‐labelled fractions by immunomagnetic activated cell sorting following the manufacturer's instructions (Stem cell Technology, Vancouver, BC, Canada). The purity of positive cells was confirmed by flow cytometry (90.7 ± 1.7%). Sca‐1^+^, Sca‐1^−^, or unsorted‐BM cells (2 × 10^6^) were directly injected into lethally irradiated (9.5 Gy) aged recipient C57BL/6 mice (20‐22 months) through the tail vein, generating Sca‐1^+^ [Y(Sca‐1^+^)‐O], Sca‐1^−^ [Y(Sca‐1^−^)‐O] and unsorted‐BM [Y(unsorted)‐O] chimaeras, respectively. Animals were randomly divided into three chimaeric groups [Sca‐1^+^, Sca‐1^−^, or unsorted‐BM cells (2 × 10^6^)] and randomly subdivided into two treatment conditions (retinal I/R and control) by an independent researcher who was blind to the experimental procedure.

### 2.2 Model of retinal ischaemia‐reperfusion injury

Retinal I/R injury was induced as previously described.^18^ Briefly, a normal saline reservoir was constructed with a 500 mL IV bottle of balanced salt solution (HBSS containing 0.1% heparin sodium) which was connected to a primary set of pre‐pierced Y‐site tubing. The Y‐site tubing connected to five 30‐gauge ½ inch needles with a 10‐inch segment of 30‐gauge tubing through a five‐valve manifold. The reservoir was hung from an IV pole extension and was elevated to maintain a height of 1.5 m which would subject the eye to hydrostatic pressure of 110 mmHg. At 3 months after BM reconstitution, mice were intubated and ventilated with 1% isoflurane mixed with oxygen. The pupils were dilated with 1% tropicamide. To induce I/R injury, the anterior chamber of each eye was cannulated with the 30‐gauge infusion needle approximately halfway between the zonule fibres and the apex of the cornea under a surgical microscope. The infusion was continued for 1 hour to induce retinal ischaemia. The microscope was used to verify that no leakage had occurred. Ocular distention was visually confirmed by observing that the eye subjected to I/R was larger than the contralateral eye. Together, the absence of leakage and the presence of ocular distention indicated a successful elevation of intraocular pressure which was also confirmed by whitening of the iris and loss of the red reflex. The needle was withdrawn and the intraocular pressure normalised, resulting in reperfusion, which was confirmed by the reappearance of the red reflex. The control mice underwent the same procedure with the needle inserted into the anterior chamber, but saline was not infused under pressure.

A schematic timetable illustrating the procedures conducted in the in vitro and in vivo studies can be found in Figure S1.

### 2.3 Statistical analysis

Analyses were performed with Prism version 6.0 software (GraphPad software Inc., San Diego, CA, USA). All values are expressed as mean ± SEM. Student's *t* test was used for two‐group comparisons. Comparisons of parameters amongst three or more groups were analysed using one‐way ANOVA for single‐factor variables followed by Tukey or two‐way ANOVA for two‐factor variables with repeated measures over time, followed by Bonferroni post‐hoc tests. Differences were considered statistically significant at *P* < 0.05. A sample size analysis was conducted to determine the appropriate sample size needed to reliably detect a significant difference between experimental groups.

## 3 RESULTS

### 3.1 BM‐derived Sca‐1^+^ cells had greater homing and differentiation capabilities after acute intraocular hypertensive injury

**Figure 1:**
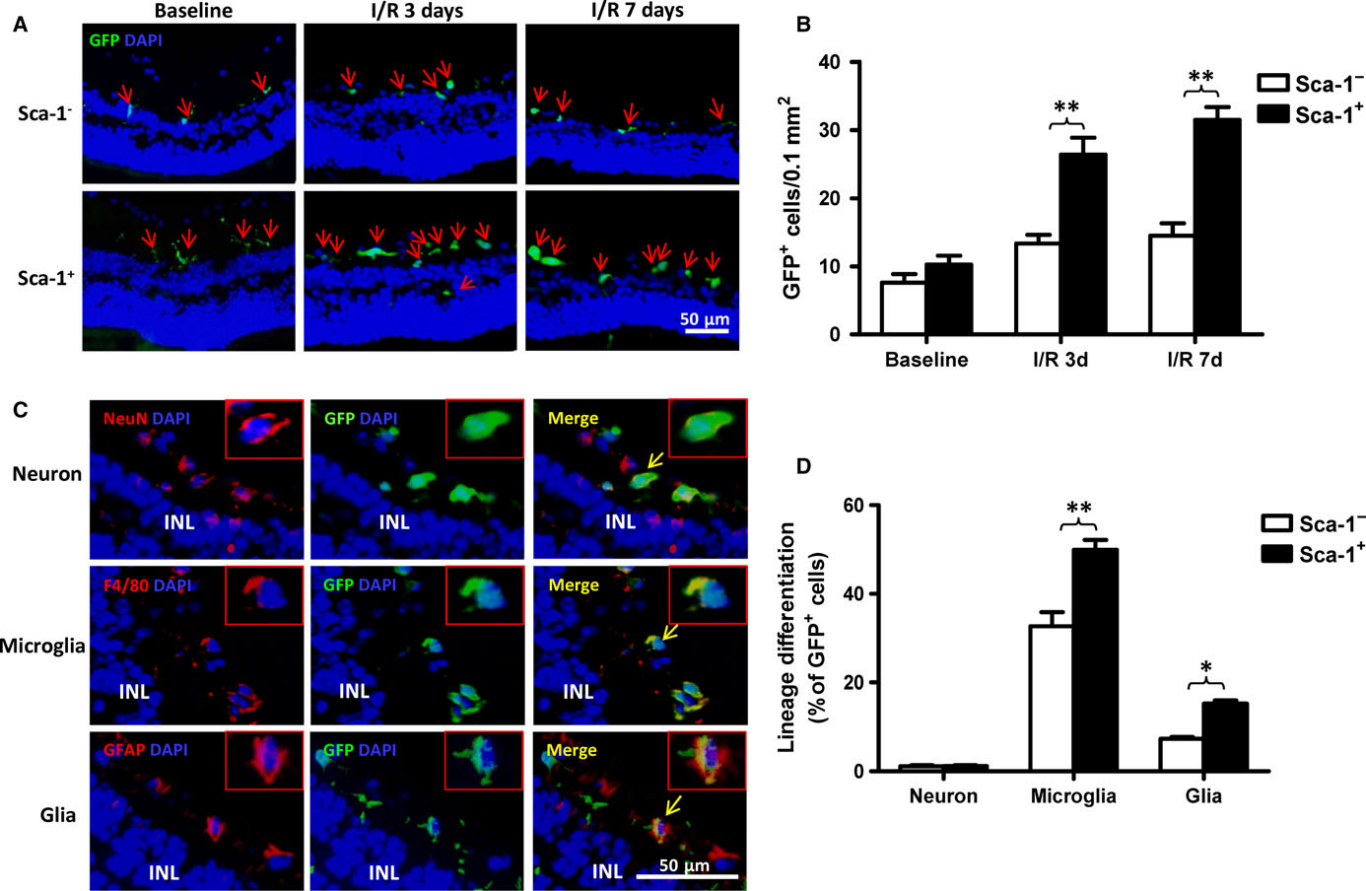
BM‐derived Sca‐1^+^ cells had greater homing and differentiation capabilities after acute ischaemia‐reperfusion injury. Bone marrow (BM) Sca‐1^+^ or Sca‐1^−^ cells from young GFP (green fluorescent protein, green, 2 × 10^6^) transgenic mice were used to reconstitute old wild‐type mice, generating Sca‐1^+^ and Sca‐1^−^ chimaeras, respectively. Acute ischaemia‐reperfusion (I/R) injury was induced 12 weeks later. Progenitor cells in the retina of recipients were evaluated 3 and 7 days post‐I/R injury. Characterisation and quantification by immunolabelling of retinal sections for GFP (A and B) and GFP/NeuN, GFP/F4/80, GFP/GFAP (Glial Fibrillary Acidic Protein) double‐positive cells (C and D). BM Sca‐1^+^ cells had greater capability to home to the retina than Sca‐1^−^ cells (n = 4/group; A and B). There was more cell differentiation into microglia (F4/80) and glia (GFAP) in Sca‐1^+^ than Sca‐1^−^ chimaeras after retinal injury (C and D; n = 4/group). INL: inner nuclear layer. Data analysis used two‐way ANOVA followed by Bonferroni post‐hoc tests for multiple comparisons (B and D). Data shown are mean ± SEM. **P < 0.01, *P < 0.05

To determine the homing capacity of the young BM Sca‐1^+^ cells to the retina of the aged recipient mice, Sca‐1^+^ [Y(Sca‐1^+^)‐O] and Sca‐1^−^ [Y(Sca‐1^−^)‐O] chimaeras were generated using young BM GFP cells. At 3 months after BM reconstitution, the reconstitution rate for Sca‐1^+^ and Sca‐1^−^ chimaeras was 48.47 ± 1.85% and 31.58 ± 3.11% in BM and 76.97 ± 1.81% and 47.76 ± 3.87% in blood, respectively. GFP expression allowed the tracking of BM‐derived cell migration into the host retina at 3 months after BM reconstitution. At baseline without injury, only a few GFP cells were found in the retina in either Sca‐1^+^ or Sca‐1^−^ chimaeras (Figure 1A). After the induction of I/R injury, more donor–derived GFP^+^ cells were found in the host retina, especially in the inner layers of the retina in both the Sca‐1^+^ and the Sca‐1^−^ chimaeras (Figure 1A). Further quantification of the GFP^+^ cells in the injured retina 3 and 7 days after injury revealed a significantly greater number of GFP^+^ cells in the Sca‐1^+^ group than the Sca‐1^−^ group, indicating better homing capability of the Sca‐1^+^ cells (Figure 1B).

Next, to evaluate the differentiation potential of the BM Sca‐1^+^ cells, immunostaining was performed to examine if the GFP^+^ cells were also positive for the neuron marker, NeuN, the microglia marker, F4/80, or the glia marker, GFAP. As shown in Figure 1C, there were GFP^+^ cells which were also positive for NeuN, F4/80 and GFAP, indicating that the homed young cells had the ability to differentiate into all three cell lineages. Quantification of the number of double‐positive cells showed that nearly 49.9 ± 4.54% of GFP^+^ cells also expressed the microglial marker, and 15.25 ± 1.45% expressed the glial marker in the Sca‐1^+^ chimaeric retina. Conversely, in the Sca‐1^−^ chimaeras, the corresponding percentages were 32.66 ± 6.45% and 7.34 ± 0.82%, respectively, indicating that more homed cells differentiated into microglia and glia in the Sca‐1^+^ than Sca‐1^−^ group (Figure 1D). On the other hand, only 1% of GFP^+^ cells expressed the neuron marker NeuN in both the Sca‐1^−^ and Sca‐1^+^ chimaeric retina with no difference between the two groups (Figure 1D).

### 3.2 Homed BM Sca‐1^+^ cells improved healing of the retina

**Figure 2:**
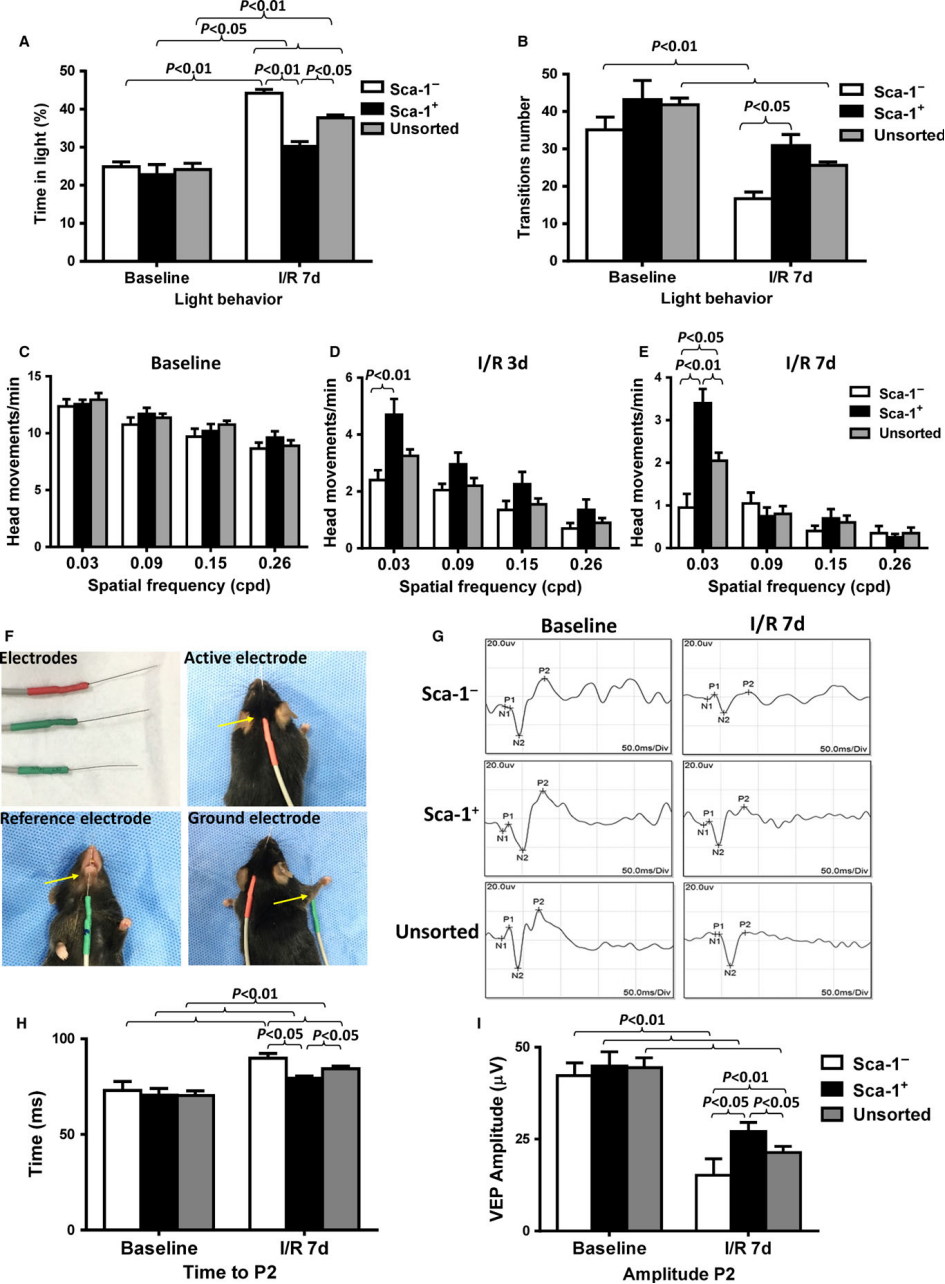
Homed BM Sca‐1^+^ cells improved healing of injured retina. The bone marrow (BM) of irradiated old wild‐type mice was reconstituted with 2 × 10^6^ young BM Sca‐1^+^, Sca‐1^−^ or unsorted cells, generating Sca‐1^+^, Sca‐1^−^, and unsorted chimaeras, respectively. Acute ischaemia‐reperfusion (I/R) injury was induced 12 weeks later. The light/dark exploration (A and B) and optomoter (C‐E) tests revealed better preservation of visual behaviour in the unsorted than Sca‐1^−^ chimaeras, and the Sca‐1^+^ chimaeras had the best preserved visual behaviour at 7 days after I/R injury (n = 10/group). (F) Flash‐visual evoked potential (VEP) was used to evaluate nerve conduction and axon potential propagation through the visual system. Flash VEP waveform latency showed significant delay in the initiation of positive peak (time to P2; G and H) and decreased amplitude of P2 (I) following visual stimulation in Sca‐1^−^ chimaeras compared to unsorted‐BM chimaeras 7 days after I/R injury (n = 6/group). However, the Sca‐1^+^ chimaeras had better preservation of visual function than both the unsorted‐BM and Sca‐1^−^ chimaeras. Data analysis used two‐way ANOVA followed by Bonferroni post‐hoc tests for multiple comparisons (A‐E, H and I). Data shown are mean ± SEM. **P < 0.01, *P < 0.05

Visual behaviour following retinal I/R injury of Sca‐1^+^, Sca‐1^−^, or unsorted‐BM chimaeric mice was evaluated using the light/dark box exploration (Figure S2) and optomotor response (Figure S3) tests. In the light/dark exploration test, mice with normal visual behaviour preferred the dark environment, and the time spent in the light compartment and the number of transitions was similar in the Sca‐1^+^, Sca‐1^−^ and unsorted‐BM chimaeras at baseline before eye injury (Figure 2A and B). After the transient increase in ocular pressure (I/R injury), all three groups of chimaeras stayed longer in the light compartment and there were fewer transitions. However, the time spent in the light compartment was significantly less for the unsorted‐BM chimaeras than the Sca‐1^−^ chimaeras, and the Sca‐1^+^ chimaeras spent the least amount of time in the light. In addition, the number of transitions was significantly greater for the Sca‐1^+^ chimaeras than the other two groups, suggesting that the Sca‐1^+^ chimaeras had better preservation of visual behaviour.

The optomotor test was evaluated by quantifying the number of head movements in the photopic condition during the rotation of the drum. Although visual function was decreased in all three groups following I/R injury, the Sca‐1^+^ chimaeras responded to lower frequencies of the drum with a greater number of head movements compared to both the unsorted‐BM and Sca‐1^−^ chimaeras at 3 and 7 days after I/R injury (Figure 2C‐E). The unsorted‐BM chimaeras produced a greater number of head movements compared to the Sca‐1^−^ chimaeras only at 7 days after I/R injury (Figure 2E).

Flash‐visual evoked potential (VEP) was used to evaluate nerve conduction and axon potential propagation through the visual system (Figure 2F). The acute elevation of intraocular pressure induced retinal damage. The most consistent component observed in flash‐VEP at baseline and 7 days after I/R injury was the second positive P2 (Figure 2G). Under all stimulus conditions, the average P2 latency response was longer compared to that at baseline in all chimaeric groups (Figure 2H). However, P2 latency after I/R injury was significantly longer in Sca‐1^−^ (89.9 ± 2.4 ms) than in unsorted‐BM (84.4 ± 1.4 ms) chimaeras. Moreover, Sca‐1^+^ chimaeras had the shortest P2 latency after I/R injury compared to both the unsorted‐BM and Sca‐1^−^ chimaeras.

The amplitude of P2 decreased significantly at 7 days after I/R injury compared with that at baseline in all three chimaeric groups (Figure 2I). However, the amplitude of P2 in Sca‐1^−^ chimaeras (15.2 ± 4.5 μv) was even lower than in unsorted‐BM chimaeras (21.3 ± 1.7 μv) at 7 days after I/R injury. The amplitude of P2 in Sca‐1^+^ chimaeras (27.1 ± 2.5 μv) was the greatest compared to the other two groups. The shorter P2 latency and the increase in P2 amplitude suggest that Sca‐1^+^ chimaeras had better preservation of visual function.

### 3.3 Homed BM Sca‐1^+^ cells reduced cellular apoptosis in the host retina

**Figure 3:**
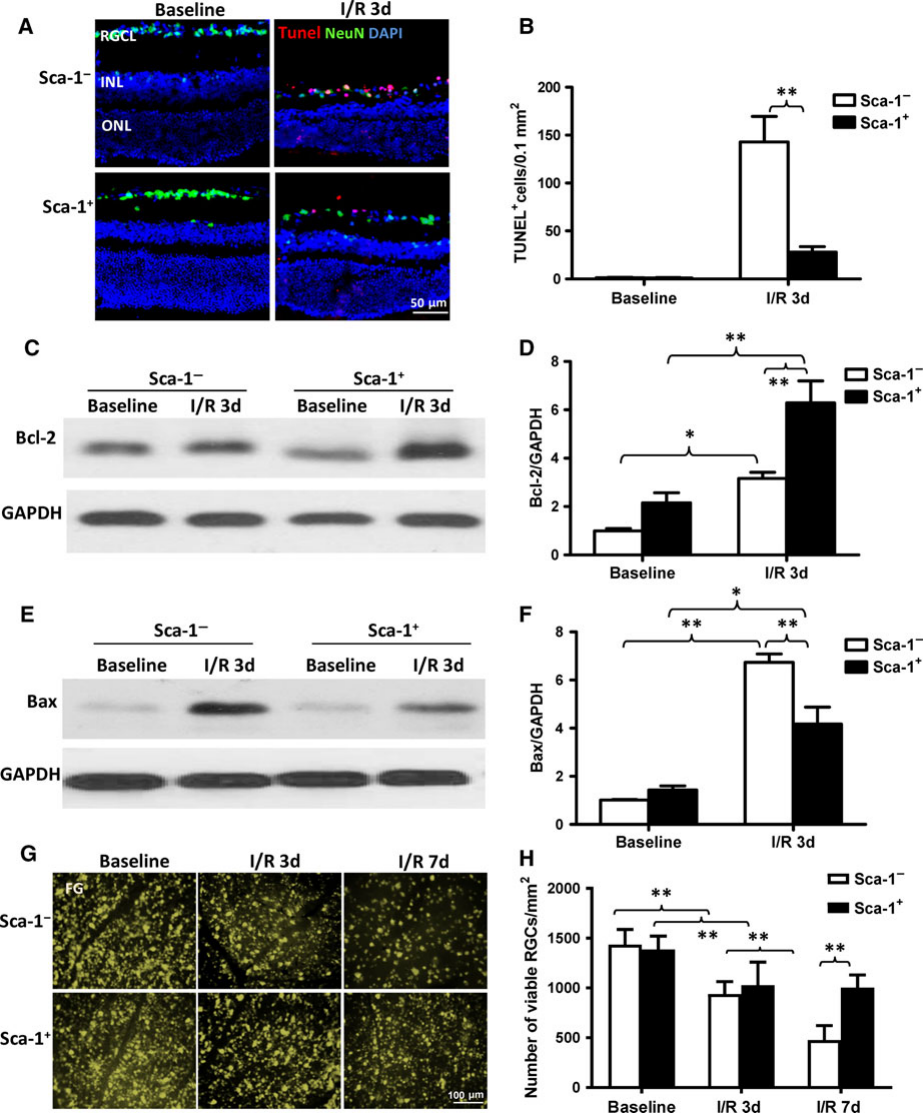
Homed BM Sca‐1^+^ cells rescued the host retina from cellular apoptosis. Cellular apoptosis was evaluated 3 days post‐ischaemia‐reperfusion (I/R) injury using Tunel staining. (A) Representative images of apoptotic cells (Tunel^+^, red) and (B) quantification of Tunel^+^ cells in Sca‐1^+^ and Sca‐1^−^ chimaeras with or without I/R injury (n = 4/group). Bcl‐2 and Bax protein expression was evaluated by Western blot. (C) Representative Western blot image and (D) quantification of Bcl‐2 protein expression in Sca‐1^+^ and Sca‐1^−^ chimaeras with or without I/R injury. GAPDH was used as a loading control (n = 4/group). (E) Representative Western blot image and (F) quantification of Bax protein expression in chimaeric mice with or without I/R injury. GAPDH used as a loading control (n = 3/group). (G) Representative images and (H) quantification of viable RGCs using FluoroGold (FG)‐labelling showed that the number of RGCs decreased at 3 and 7 days after reperfusion. However, the number of RGCs that survived was significantly greater in the retinas of Sca‐1^+^ than Sca‐1^−^ chimaeric mice at 3 and 7 days after I/R injury. RGC: retinal ganglion cell; RGCL: retinal ganglion cell layer; INL: inner nuclear layer; ONL: outer nuclear layer. Data analysis used two‐way ANOVA followed by Bonferroni post‐hoc tests for multiple comparisons (B, D, F and H). Data shown are mean ± SEM. **P < 0.01, *P < 0.05

To further elucidate the possible mechanisms responsible for the repair of the aged retina after I/R injury in the Sca‐1^+^ and Sca‐1^−^ chimaeric mice, TUNEL staining was performed at 3 days after injury and more apoptotic cells were observed mostly in the RGC layer of the retina of Sca‐1^−^ than Sca‐1^+^ chimaeric mice (Figure 3A and B). The protein expression of Bcl2, the downstream protective mediator of cellular apoptosis, increased in both groups 3 days post‐I/R injury, but was significantly greater in the Sca‐1^+^ than Sca‐1^−^ chimaeric retina (Figure 3C and D). Conversely, the protein expression of Bax, the downstream mediator of cell apoptosis, was significantly lower in Sca‐1^+^ than Sca‐1^−^ chimaeras at 3 days after I/R injury (Figure 3E and F). In addition, FluoroGold‐labelling of viable RGCs demonstrated that the number of RGCs decreased at 3 and 7 days after reperfusion. However, the number of viable RGCs was significantly greater in the retina of the Sca‐1^+^ than Sca‐1^−^ group at 3 and 7 days after I/R injury (Figure 3G and H). Collectively, these results indicate that homed BM Sca‐1^+^ cells protected the host retina from cellular apoptosis.

### 3.4 Homed BM‐derived Sca‐1 cells improved repair of the retina through the FGF2 pathway

**Figure 4:**
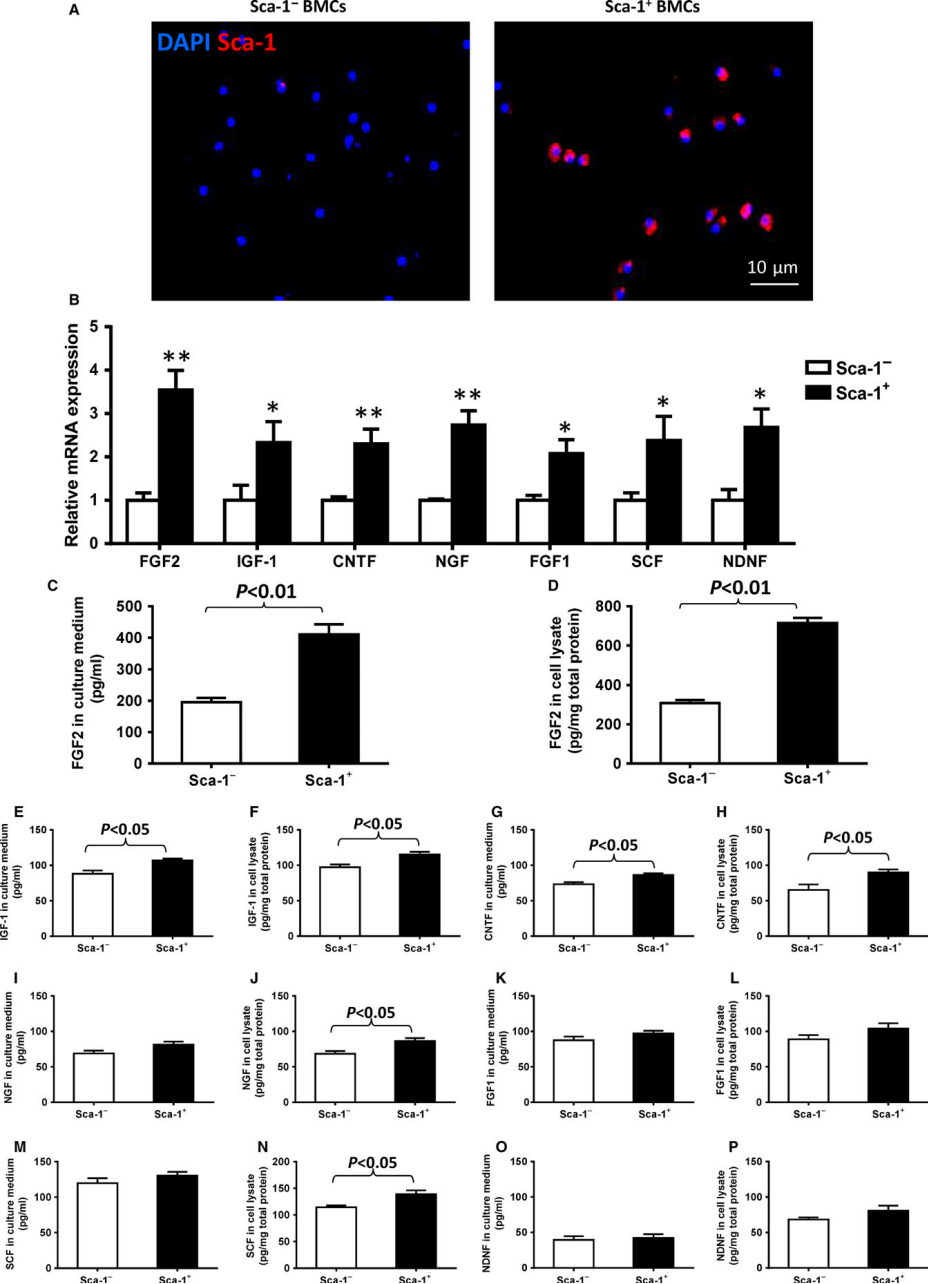
Differential expression profile of growth factors between BM‐derived Sca‐1^+^ and Sca‐1^−^ subset of cells. (A) Immunolabelling confirmed the phenotype of bone marrow (BM)‐derived Sca‐1^+^ and Sca‐1^−^ cells. (B) The expression profiles of growth factors were determined by RT‐qPCR. The mRNA levels of FGF2 (fibroblast growth factor 2), IGF‐1 (insulin‐like growth factor 1), CNTF (ciliary neurotrophic factor), NGF (nerve growth factor), FGF1 (fibroblast growth factor 1), SCF (stem cell factor) and NDNF (neuron‐derived neurotrophic factor) were significantly higher in Sca‐1^+^ than Sca‐1^−^ cells. Amongst these growth factors, the mRNA level of FGF2 increased most dramatically in the BM Sca‐1^+^ cells relative to the Sca‐1^−^ cells. (C‐P) After 48 hours of culture, FGF2, IGF‐1, CNTF, NGF, FGF1, SCF, and NDNF protein levels in the culture medium and cell lysate were determined by ELISA. FGF2 protein level was significantly greater in the medium and cell lysate of BM Sca‐1^+^ than Sca‐1^−^ cells (n = 4/group). Data analysis used un‐paired *t* test. Data shown are mean ± SEM. *P < 0.05, **P < 0.01

Recent studies demonstrated that growth factors have beneficial effects on cell survival in the retina under pathological conditions.^19^ To elucidate the possible factors responsible for the BM Sca‐1^+^ cell‐mediated protection from retinal injury, BM Sca‐1^+^, and Sca‐1^−^ cells were successfully isolated from young donor mice (Figure 4A) and differential expression profiles of growth factors for the BM‐derived Sca‐1^+^ and Sca‐1^−^ subset of cells were compared using RT‐qPCR. As shown in Figure 4B, the mRNA levels of FGF2, insulin‐like growth factor 1 (IGF‐1), ciliary neurotrophic factor (CNTF), nerve growth factor (NGF), FGF1, stem cell factor (SCF), and neuron‐derived neurotrophic factor (NDNF) were significantly higher in Sca‐1^+^ than Sca‐1^−^ cells and the mRNA level of FGF2 increased most dramatically in the Sca‐1^+^ cells. To further clarify whether these factors were secreted from the migrating Sca‐1 cells, BM Sca‐1 cells were cultured for 48 hours and the level of these factors in the cell culture supernatant and cell lysate was measured using ELISA (Figure 4C‐P). As shown in Figure 4C and D, there was significantly more FGF2 protein in both the medium and lysate from Sca‐1^+^ cells compared to Sca‐1^−^ cells. This result clearly indicates that FGF2 was directly secreted from the Sca‐1 cells. However, only slight increases in cytokine levels of IGF‐1 and CNTF (both in the media and in cell lysate, Figure 4E‐H) were detected in the BM Sca‐1^+^ cells relative to the Sca‐1^−^ cells. There were no differences in NGF (Figure 4I), FGF1 (Figure 4K), SCF (Figure 4M), or NDNF (Figure 4O) levels between the culture media from BM Sca‐1^+^ and Sca‐1^−^ cells. There were small increases in NGF (Figure 4J) and SCF (Figure 4N) levels in the cell lysate from BM Sca‐1^+^ relative to Sca‐1^−^ cells.

**Figure 5:**
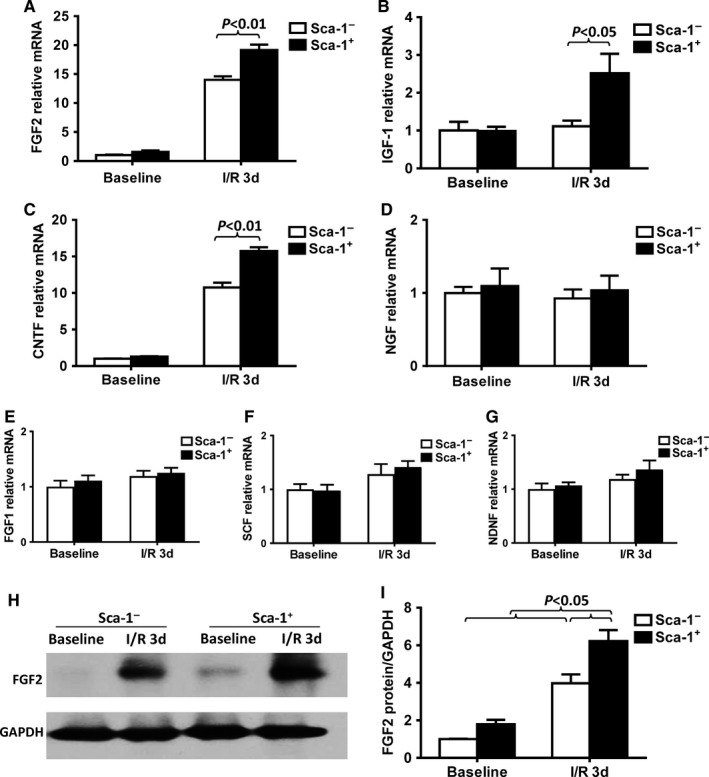
Homed BM‐derived Sca‐1^+^ cells prevented retinal injury through activation of FGF2 pathway. BM Sca‐1^+^ and Sca‐1^−^ cells from young GFP (green fluorescent protein, green, 2 × 10^6^) mice were used to reconstitute irradiated old wild‐type mice, generating Sca‐1^+^ and Sca‐1^−^ chimaeras. Acute ischaemia‐reperfusion (I/R) injury was induced 12 weeks later. (A‐G) The expression profiles of growth factors in the retinas of Sca‐1^+^ and Sca‐1^−^ chimaeras with or without ischaemia‐reperfusion (I/R) injury were determined by RT‐qPCR. The mRNA levels of FGF2 (fibroblast growth factor 2), IGF‐1 (insulin‐like growth factor 1) and CNTF (ciliary neurotrophic factor) were significantly higher in Sca‐1^+^ than Sca‐1^−^ chimaeras. (H‐I) FGF2 protein expression, as assessed by Western blot, was significantly higher in the retinas of Sca‐1^+^ than Sca‐1^−^ chimaeras 3 days after I/R injury. NGF: nerve growth factor, SCF: stem cell factor, NDNF: neuron‐derived neurotrophic factor. n = 4/group; Data analysis used two‐way ANOVA followed by Bonferroni post‐hoc tests for multiple comparisons (A‐G and I). Data shown are mean ± SEM. *P < 0.05, **P < 0.01

The expression profiles of growth factors in the retinas of Sca‐1^+^ and Sca‐1^−^ chimaeric mice with or without I/R injury were examined at the mRNA and protein levels. The mRNA levels of FGF2, IGF‐1, and CNTF were significantly higher in the Sca‐1^+^ than Sca‐1^−^ chimaeras at 3 days post‐I/R injury (Figure 5A‐C), though there was no significant difference in the mRNA level of NGF, FGF1, SCF, and NDNF mRNA between the two groups (Figure 5D‐G) at 3 days. RT‐qPCR analysis revealed that the most dramatic increase was in the FGF2 mRNA level in both Sca‐1^+^ and Sca‐1^−^ chimaeras compared to their baseline values (Figure 5A). In agreement with the mRNA expression pattern, FGF2 protein expression, as assessed by Western blot, was significantly higher in the retinas of the Sca‐1^+^ than Sca‐1^−^ chimaeras 3 days after I/R injury (Figure 5H and I). These results suggest that FGF2 may play a pivotal role in mediating the protective effect of BM‐derived Sca‐1 cells on retinal I/R injury.

### 3.5 In vitro co‐culture of BM‐derived Sca‐1 cells with a retina explant decreased retinal cell apoptosis

**Figure 6:**
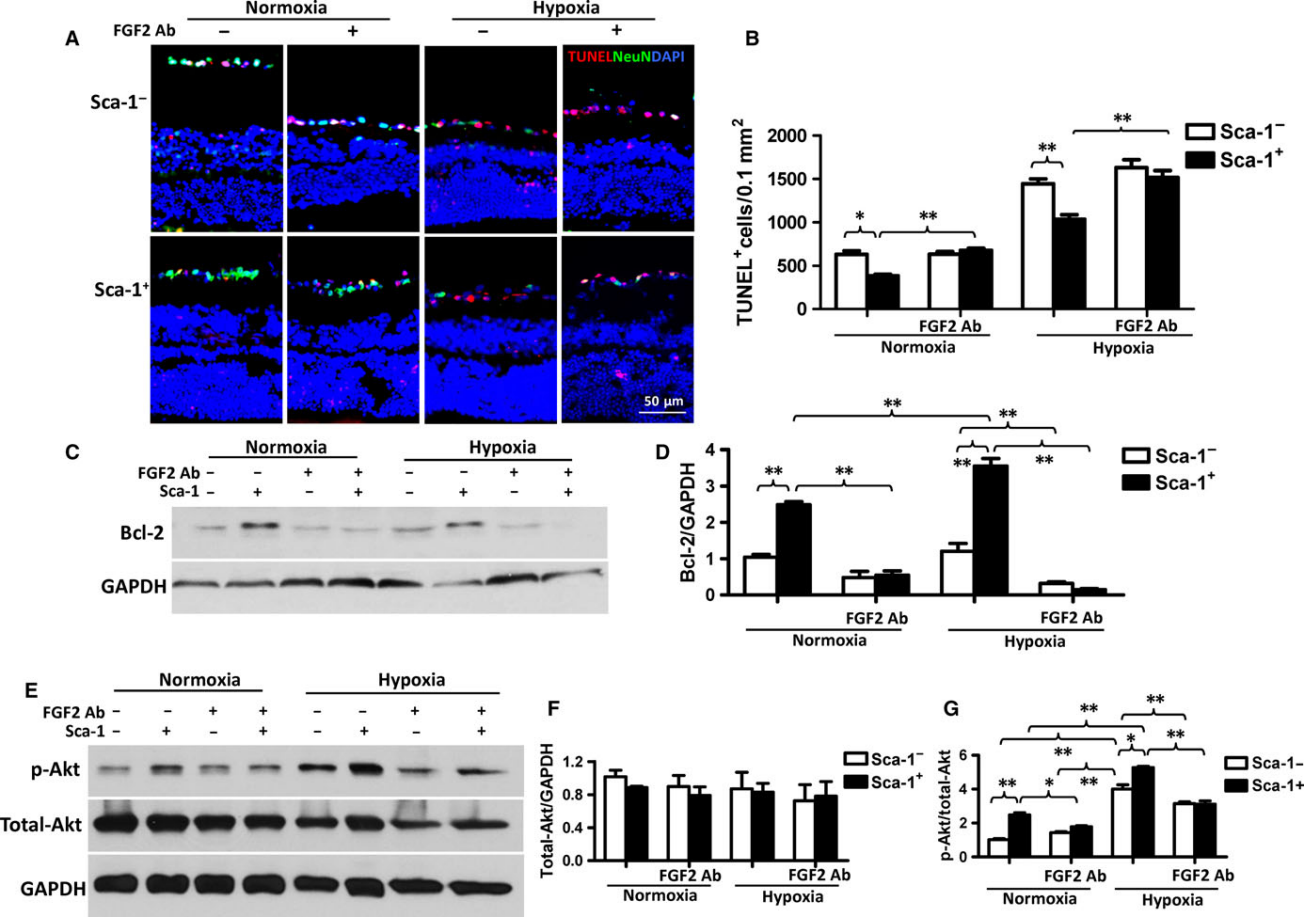
In vitro co‐culture of BM‐derived Sca‐1^+^ cells with retina explants decreased retinal cell apoptosis. Retina explants were co‐cultured with BM‐derived Sca‐1^+^ or Sca‐1^−^ cells under normoxia and hypoxia conditions. (A) Representative images of apoptotic cells (Tunel^+^, red) and NeuN^+^ (green) neurons. Nuclei were stained with DAPI. (B) Co‐culture with BM Sca‐1^+^ cells protected the retina cells from hypoxia‐induced apoptosis. However, this protective effect was lost after application of an FGF2 (Fibroblast Growth Factor 2) neutralising antibody (Ab, n = 6/group). (C and D) Bcl‐2 protein expression was significantly greater in retina explants co‐cultured with BM Sca‐1^+^ than Sca‐1^−^ cells under both normoxia and hypoxia conditions as assessed by Western blot. However, this effect was blocked by an FGF2 antibody (Ab). GAPDH was used as a loading control (n = 3/group). (E‐G) Total Akt and p‐Akt (phospho Ser473‐Akt) expression was assessed by Western blots. The ratio of p‐Akt/total Akt expression was significantly greater in retina explants co‐cultured with BM Sca‐1^+^ than Sca‐1^−^ cells under both normoxia and hypoxia conditions. The ratio of p‐Akt/total Akt expression was significantly decreased after the application of an FGF2 Ab (n = 3/group). Data analysis used two‐way ANOVA followed by Bonferroni post‐hoc tests for multiple comparisons (B, D, F and G). Data shown are mean ± SEM. **P < 0.01, *P < 0.05

To further confirm that BM Sca‐1 cells protected the host retina from cellular apoptosis and also to further elucidate the role of FGF2 in mediating the protective effect of BM‐derived Sca‐1 cells on retinal I/R injury, we utilised an in vitro co‐culture model using an FGF2 neutralising antibody. BM‐derived Sca‐1^+^ or Sca‐1^−^ cells were co‐cultured on the inner retinal (vitreous) surface of organotypic retinal explants under normoxic and hypoxic conditions in the presence and absence of an FGF2 neutralising antibody for 48 hours. Quantification of cell apoptosis within the RGC layer of retinal explants demonstrated that co‐culture with BM Sca‐1^+^ cells was associated with a significant attenuation of hypoxia‐induced apoptosis compared to Sca‐1^−^ cell co‐culture (Figure 6A and B). This result demonstrates the protective effect of BM Sca‐1^+^ cells on host retinal cells. However, this protective effect was lost after the application of an FGF2 neutralising antibody (Figure 6A and B). Bcl‐2 protein expression was found to be significantly increased in retina explants co‐cultured with BM Sca‐1^+^ cells compared to Sca‐1^−^ cells under both normoxic and hypoxic conditions. However, this effect was blocked by an FGF2 antibody (Figure 6C and D). Total Akt and p‐Akt (phospho Ser473‐Akt) further confirmed the activation of the FGF2 pathway. The ratio of p‐Akt/total Akt expression was significantly higher in retina explants co‐cultured with BM Sca‐1^+^ than Sca‐1^−^ cells under both normoxic and hypoxic conditions. However, the ratio of p‐Akt/total Akt expression was significantly decreased after the application of an FGF2 antibody (Figure 6E‐G). These data indicate that FGF2 signalling plays a predominant role in the anti‐apoptotic response of BM Sca‐1 cells by promoting activation of Akt. After FGF2 was blocked, the activation of the Akt pathway was also blocked, and in turn the beneficial effects of the Sca‐1^+^ cells were lost.

### 3.6 Blocking FGF2 pathway reversed the effect of BM‐derived Sca‐1 cells on retinal injury in vivo

**Figure 7:**
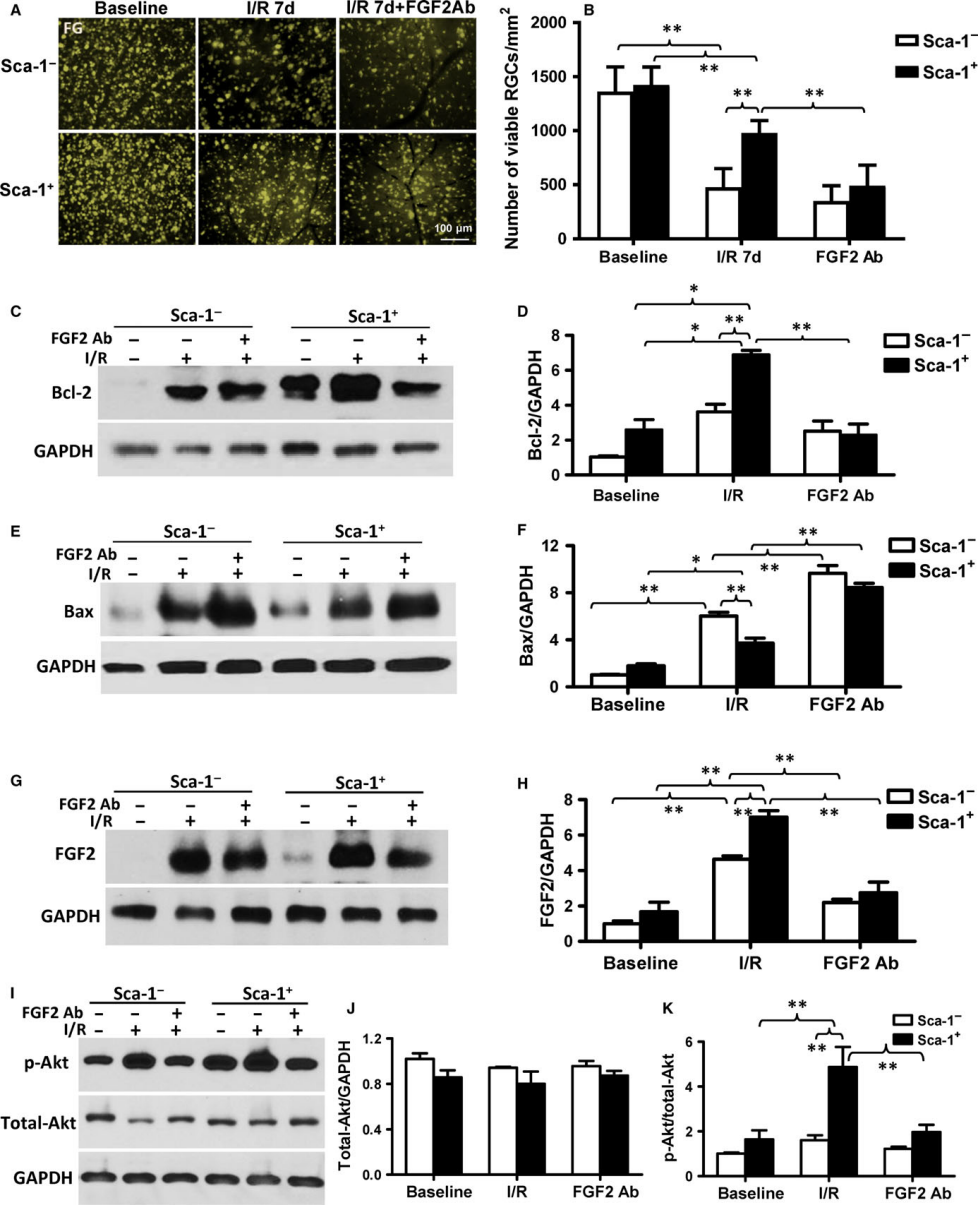
Blocking the FGF2 pathway reversed the effect of BM Sca‐1^+^ cells on retinal injury in vivo. The bone marrow (BM) of irradiated old wild‐type mice was reconstituted with 2 × 10^6^ BM Sca‐1^+^ or Sca‐1^−^ cells from young GFP (green fluorescent protein) transgenic donors, generating Sca‐1^+^ and Sca‐1^−^ chimaeras, respectively. Acute ischaemia‐reperfusion (I/R) was induced 12 weeks later. The number of RGCs that survived was determined using FluoroGold‐(FG) labelling. (A) Representative images and (B) quantification of viable RGCs in Sca‐1^+^ and Sca‐1^−^ chimaeras at baseline, 7 days after I/R injury, and in the presence and absence of an FGF2 (Fibroblast Growth Factor 2) neutralising antibody (Ab, n = 6/group). Bcl‐2 (C and D, n = 3/group) and Bax (E and F, n = 4/group) protein expression was evaluated by Western blot and GAPDH was used as a loading control. (G and H) FGF2 protein expression, as assessed by Western blot, was significantly greater in the retinas of Sca‐1^+^ than Sca‐1^−^ chimaeras 3 days after I/R injury. However, FGF2 protein expression significantly decreased with the addition of an FGF2 Ab (n = 3/group). (I‐K) Total Akt and p‐Akt (phospho Ser473‐Akt) expression was assessed by Western blot. The ratio of p‐Akt/total Akt expression was significantly greater in the retinas of Sca‐1^+^ than Sca‐1^−^ chimaeras 3 days after I/R injury. The ratio of p‐Akt/total Akt expression was significantly decreased after application of an FGF2 Ab (n = 4/group). Data analysis used two‐way ANOVA followed by Bonferroni post‐hoc tests for multiple comparisons (B, D, F, H, J and K). Data shown are mean ± SEM. **P < 0.01, *P < 0.05

To further confirm the role of FGF2 in mediating the protective effect of BM‐derived Sca‐1 cells on retinal I/R injury in vivo, we injected an FGF2 neutralising antibody into the vitreous of the Sca‐1^+^ and Sca‐1^−^ chimaeric mice after the onset of retinal ischaemia and the mice were sacrificed 3 and 7 days later. FluoroGold‐labelling of viable RGCs revealed that significantly more RGCs survived in the retina of Sca‐1^+^ compared to Sca‐1^−^ chimaeric mice (Figure 7A and B). However, the number of viable RGCs decreased significantly in Sca‐1^+^ chimaeras when an FGF2 neutralising antibody was added compared to Sca‐1^+^ chimaeras without the antibody, indicating that the FGF2 neutralising antibody partially reversed the protective effect of BM‐derived Sca‐1 cells. Bcl‐2 (Figure 7C and D) and Bax (Figure 7E and F) protein expression was evaluated in the Sca‐1^+^ and Sca‐1^−^ chimaeras at baseline, after I/R injury, and after I/R injury in the presence of an FGF2 antibody. The protein level of Bcl‐2 decreased significantly in the retinas of Sca‐1^+^ chimaeras whereas the protein level of Bax increased significantly after the application of an FGF2 neutralising antibody compared to the Sca‐1^+^ chimaeras without the antibody. The FGF2 protein expression was significantly higher in the retinas of Sca‐1^+^ than Sca‐1^−^ chimaeras 3 days after I/R injury. However, FGF2 protein expression was significantly decreased with an FGF2 antibody (Figure 7G and H). Finally, to further confirm activation of the FGF2 pathway, total Akt and p‐Akt (phospho Ser473‐Akt) expression were measured (Figure 7I‐K). The ratio of p‐Akt/total Akt expression was significantly higher in the retinas of Sca‐1^+^ than Sca‐1^−^ chimaeras 3 days after I/R injury. However, the ratio of p‐Akt/total Akt expression was significantly decreased after application of an FGF2 antibody. Together, these results were similar to those observed with the in vitro co‐culture model: BM Sca‐1^+^ cells protected the host retina cells from retinal damage induced by I/R injury through activation of the FGF2/Bcl2/Akt pathway.

## 4 DISCUSSION

In the current study, we investigated the effects of young BM‐derived Sca‐1^+^ stem cells on the functional repair of the aged retina after the induction of I/R injury. We showed that after BM reconstitution with young Sca‐1^+^ or Sca‐1^−^ cells, there was more mobilisation, homing and differentiation of BM‐derived Sca‐1^+^ cells in the aged retina after increased intraocular pressure‐induced retinal ischaemia. These homed BM Sca‐1^+^ cells protected the host retina from cellular apoptosis through increased FGF2 expression. The subsequent activation of the Akt pathway increased Bcl2 expression and decreased Bax expression. All of these responses contributed to the neuroprotective effects following increased intraocular pressure‐induced retinal ischaemic injury and improved healing of the injured retina. The retinal and optic nerve damage associated with I/R is markedly worse in older individuals.^2^, ^3^, ^4^, ^20^ These investigations suggest that the loss of BM stem cell function with age could contribute to the impairment in retina repair and regeneration after injury. Rejuvenation of aged BM by reconstitution with young Sca‐1^+^ cells reduced the functional deterioration following retinal injury. Pressure injury stimulates the release of neurotransmitters which contribute to apoptosis of neuronal cells and active BMSCs protect them from retinal apoptosis.^20^ These studies provide preliminary evidence for a new treatment strategy to mitigate the effects of increased intraocular pressure injury.

As reported in previous studies, normally sighted animals showed an aversion to light in the light/dark exploration behavioural test.^21^, ^22^ Here we showed that the Sca‐1^−^ chimaeras failed to demonstrate light aversion and reduced their movements in the majority of light/dark testing paradigms compared to the unsorted‐BM chimaeras after retinal I/R injury. However, the Sca‐1^+^ chimaeras showed a robust aversion to light and more movements compared to both the Sca‐1^−^ and unsorted‐BM chimaeras. Additionally, these results were consistent with those from the optomotor response test, which was used to evaluate visual defects in this mouse model.^23^, ^24^ Most animals compensate for a globally moving environment by turning their head to stabilise the images on the retina. An involuntary head movement was recorded as an oculomotor response, which offered a simple and rapid method to assess visual acuity. In our experiments, the Sca‐1^−^, unsorted‐BM, and Sca‐1^+^ chimaeric animals produced an optomotor response from 0.03 to 0.26 cpd before retina impairment. After retinal damage (by increased intraocular pressure), the Sca‐1^−^ and unsorted‐BM chimaeras displayed almost no optomotor response at those same spatial frequencies of the grating test. However, the Sca‐1^+^ chimaeras still demonstrated head responses at the lowest grating.

The VEP has been employed to identify and monitor glaucomatous nerve damage^25^, ^26^ and the components of the flash‐VEP are reduced in glaucoma.^27^ Our findings from the flash‐VEP recording showed that both amplitude and latency of the main P2 component were affected by I/R injury. However, the longer P2 latency and lower amplitude observed in the Sca‐1^−^ chimaeras indicated that their visual function was worse than both the unsorted‐BM and Sca‐1^+^ chimaeras. The Sca‐1^+^ chimaeras had the best preservation of visual function relative to both the unsorted‐BM control and Sca‐1^−^ chimaeras.

Consistent with this notion, we identified some of the cellular mechanisms which may explain the protective effects of the BM Sca‐1^+^ cells on visual function after injury. The inhibition of FGF2 significantly suppressed the activation of Akt signalling and exacerbated retinal ischaemic damage and RGC death. Sca‐1^+^ chimaeric mice had better preservation of visual function than Sca‐1^−^ chimaeras, suggesting that BM Sca‐1^+^ cells enhanced the regenerative capacity of the aged retina through homing and differentiation which in turn stimulated paracrinal support.

Bone marrow is a heterogeneous compartment that hosts multiple cell types and precursors. The mechanisms responsible for the regenerative effects of BM cells are still controversial. Evidence has accumulated that circulating BMSCs play an important role in keeping a balance amongst the stem cell populations in various anatomic areas of the body.^10^ However, aging leads to a gradual decline in stem cell function which reduces their regenerative potential, a decrease in immune function and the loss of homoeostatic control which may contribute to a variety of pathologic processes.^15^, ^28^, ^29^ We found that Sca‐1^+^ cells from young donors showed superior capacity and greater efficiency than Sca‐1^−^ cells to home to and integrate into the aged retina to repopulate the stem cell population in the aged eye after injury. These Sca‐1^+^ cells migrated to the retina, expressed microglial and glial markers and improved retinal response to injury. Other studies have also identified the ability BMSCs, including haematopoietic stem cells, to cross lineage boundaries and to express tissue‐specific proteins in the retina and contribute to retinal repair after damage.^11^, ^13^, ^30^, ^31^, ^32^, ^33^

Amongst the circulating BMSCs that migrated to the retina, BM‐derived microglia in the retina are of particular interest since they have been reported to be recruited by apoptotic neuronal cells in the retina.^33^, ^34^ Microglia, monocyte‐derived macrophages, and other innate immune cell types play an important role in central nervous system (CNS) homoeostasis during development, adulthood, and aging.^35^ A proper balance between the inflammatory responses of these innate immune cells is essential for efficient tissue repair, and immune modulation may be an effective way to promote repair and enhance regenerative therapies.^7^, ^36^ Microglia express many versatile receptors^37^ and are considered to be the main recipient of various signals from the degenerating neurons. In a study conducted by London et al,^38^ monocyte‐derived macrophages were found to infiltrate the retina of mice after injury. Inhibition of this infiltration resulted in reduced survival of RGCs and diminished numbers of proliferating retinal progenitor cells (RPCs) in the ciliary body. Enhancement of the circulating monocyte pool led to increased RGC survival and RPC renewal. This study suggests that microglia may play an important role as unique endogenous neuroprotective agents in treating retinal neuropathies as well as other CNS pathologies that involve neuronal loss. Moreover, in a retinal degeneration model, activated microglia interacted with Müller glial cells to control neurotrophic factor production and improve cell survival.^39^ Indeed, we also found that BM Sca‐1^+^ cells differentiated into microglia after injury. These differentiated Sca‐1^+^ cells may also act as a unique endogenous neuroprotective agent or they may interact with Müller glial cells to control neurotrophic factor production to improve host retinal cell survival.

Delivery of young BM cells to the injured eye may be difficult. In this study, we describe a new and very effective means to deliver these retinal‐protective cells. Rather than direct injection, we reconstituted the BM with these young cells and then employed the normal method of engraftment into the retina. This preliminary study provided proof of principle evidence that this approach is effective. Future studies will need to determine how long after injury BM reconstitution remains effective at regenerating the injured retina.

In this study, we also identified some of the potential mechanisms responsible for the regenerative effects of the young BM Sca‐1^+^ cells. This study demonstrated that BM Sca‐1^+^ cells expressed more growth factors than BM Sca‐1^−^ cells. Consistent with this finding, other studies have shown a significantly higher expression of BDNF and FGF2 mRNA in the Sca‐1^+^Lin^−^CD45^−^‐derived BM stem cell population which could enhance survival and neurite outgrowth of dopaminergic neurons.^40^ When we compared a variety of neurotrophic factors expressed by the BM‐derived Sca‐1^+^ and Sca‐1^−^ stem cells, the mRNA of FGF2, IGF‐1, CNTF, NGF, FGF1, SCF, and NDNF were significantly higher in the Sca‐1^+^ subset. Amongst all these growth factors, FGF2 was identified as a particularly potent neuroprotectant molecule with the highest expression in BM Sca‐1^+^ cells. FGF2 has previously been described as a neurotrophic factor,^41^ and is expressed throughout the retina and CNS. FGF2 also exerts a direct neuroprotective effect and can stimulate progenitor cell formation in the retina.^42^, ^43^, ^44^ Moreover, it was reported that a reduction in FGF2 expression could contribute to the age‐related impaired function of human mesenchymal‐derived progenitor cells.^45^ Our data showed robust expression of the FGF2 protein in the retina of Sca‐1^+^ chimaeras in response to acute I/R injury that was greater than that seen in Sca‐1^−^ chimaeras and was associated with decreased cellular apoptosis and greater neuronal preservation in the Sca‐1^+^ chimaeras. Although the evidence supports that FGF2 is the major factor involved in the BM Sca‐1^+^ cell‐mediated protection of the retina from I/R injury, possible effects from other cytokines secreted by BM Sca‐1 cells cannot be completely excluded. In addition, effective replacement of BM stem/progenitor cells provided a continuous source of homing of the cells to the site of the injury in a temporal and spatial manner when the repair was needed. Timing of the application and dose of FGF2 needs to be established. Also, it would be technically challenging to continuously supply FGF2 to the retina at the right time and the right place. Reconstituting the BM with these young stem/progenitor cells and then employing the normal method of engraftment into the retina still provided an effective approach to protect against retinal injury.

Associated with the increase in FGF2 protein in Sca‐1^+^ chimaeras, the anti‐apoptotic protein Bcl2 was up‐regulated and the apoptotic protein Bax was down‐regulated. Our data suggest that the improved FGF2 expression along with the homing of young BM‐derived Sca‐1^+^ cells may potentiate the regenerative capacity of the aged retina after acute I/R damage. Indeed, Akt, the central mediator for cell survival and tissue regeneration was activated in response to the elevation of the FGF2 protein in the Sca‐1^+^ chimaeric retina. Akt is involved in cellular survival pathways by inhibiting apoptotic processes. Akt also positively regulates some transcription factors to allow expression of pro‐survival genes. Akt phosphorylates and activates the expression of caspase inhibitors,^46^ and also stimulates the recruitment of Bcl2 to promote cell survival.^47^ Here we showed that BM Sca‐1^+^ cells activated Akt possibly through the FGF2 pathway, thereby protecting retinal cells against acute I/R injury. By utilising a neutralising antibody, we confirmed that FGF2 signalling is necessary for the neuroprotective effects of BM‐derived Sca‐1^+^ cells.

## 5 CONCLUSION

We identified a subset of BM‐derived Sca‐1 cells that contributes to aged retinal repair and restoration of visual function after acute I/R injury by both a direct contribution to cellular components and by supporting endogenous repair. Young Sca‐1^+^ cells in aged recipient BM can home to the retina and repopulate the retinal stem cells and reduce cellular apoptosis after acute I/R injury in aged mice. FGF2 and its signalling pathway were demonstrated to play an important role in Sca‐1^+^ cell‐mediated retinal neuroprotection. A deeper understanding of the neuroprotective effects of BM‐derived Sca‐1^+^ cells on functional recovery, wound repair, and tissue maintenance may facilitate the development of effective therapies for progressive neurodegeneration.

## CONFLICT OF INTEREST

The authors confirm that there are no conflicts of interest.

## AUTHOR CONTRIBUTION

Z‐BS, JW and G‐QD contributed to the design of the study, analysis and interpretation of the data, and wrote the manuscript; H‐FS, SH, JL and J(un)W contributed to the collection of data and data analysis and interpretation; S‐HL contributed to conception and design, data analysis and interpretation, manuscript writing, and final approval of manuscript; RDW contributed to manuscript writing and final approval of manuscript. H‐PY and R‐KL contributed to conception and design, manuscript writing, and final approval of manuscript.
