# Supplementary material for: Young bone marrow Sca‐1 cells protect aged retina from ischaemia‐reperfusion injury through activation of FGF2
Source: J Cell Mol Med. 2018 Sep 25;22(12):6176–89. doi: 10.1111/jcmm.13905 (PMC6237572; doi:10.1111/jcmm.13905)
Supplement: Supplementary file 1 [file JCMM-22-6176-s001.docx]

**Supplemental Information**

**for**

**Young Bone Marrow Sca-1 Cells Protect Aged Retina from Ischemia-Reperfusion Injury through Activation of FGF2**

Zhengbo Shao^1, 2†^, Jie Wu^1 ,2†^, Guoqing Du^1, 2†^, Huifang Song^2,4^, Shu-hong Li^2^, Sheng He^2,4^, Jiao Li^2,5^, Jun Wu^2^, Richard D. Weisel^2, 3^, Huiping Yuan^1*^, Ren-Ke Li^2,3*^

^1^Department of Ophthalmology, the Second Affiliated Hospital of Harbin Medical University, Harbin, China; Research Institute, Second Affiliated Hospital of Harbin Medical University, Harbin, China; Laboratory of Medical Genetics, Harbin Medical University, Harbin, China

^2^Division of Cardiovascular Surgery, Toronto General Hospital Research Institute, University Health Network, Toronto, ON, Canada

^3^Division of Cardiac Surgery, Department of Surgery, University of Toronto, Toronto, ON, Canada

^4^Shanxi Medical University, Taiyuan, China

^5^Department of Cardiology, Second Affiliated Hospital of Guangzhou Medical University, Guangzhou, China.

^†^These authors contributed equally to this work.

**Supplemental Materials and Methods**

***Light/dark box exploration task***

We investigated light-mediated visual behavior using a light/dark box exploration paradigm [1]. As previously described, the light–dark exploration test was carried out in a light/dark box consisting of a dark and an illuminated chamber of equal size (22.5×20×16 cm[L×W×H]). Animals could move freely between the two chambers through a 4×5 cm aperture located at the midpoint of the black wall separating the two compartments (Supplemental Figure 2). The light/dark test was based on rodents’ unconditioned preference for darker compared to brighter areas and an increase in the number of times that they were in the light chamber suggested diminished visual acuity. All tests were conducted in a quiet, darkened room in the Rodent Behavior Analysis Core Facility at Toronto General Hospital Research Institute. To minimize the effect of rodents’ tendency to be more anxious in novel environments, the animals were habituated to the test arena prior to the start of actual testing. The pre-test habituation phase involved exposing animals to the light/dark box 2 to 3 times (i.e., 2 to 3 trials) per day, with the ambient light level of the light compartment within a range of low lighting conditions (1 to 100 lux). Nominal testing began when a stable level of temporal distribution between the two compartments was reached at all light intensities used during the habituation phase. Mice were dark adapted in complete darkness for more than 12h prior to testing. Animals were placed into and removed from the test chamber under 1-lux lighting conditions. The dark chamber was surrounded by light-impermeable fabric to ensure complete darkness. The light chamber was lit with a light consisting of 1000 lux using a custom LED array suspended and centered above the light emitting chamber. The mouse was initially placed in the dark chamber. Its activity was quantified over a 5 min period by determining the number of times it entered the light chamber, the time spent in this chamber, and the number of transitions between the two chambers. A mouse was deemed to have entered a chamber when all four paws had crossed into that chamber. Each animal was tested 5 times per trial with an inter-trial interval of 10 min. Ten animals from each experimental group were tested. Data were calculated manually by viewing the video recording and averaging the data from each trial.

***Optomotor response task***

In both scotopic and photopic conditions, the rotation of a grating was found to elicit head movements in mice and the highest spatial frequency eliciting this optomotor response provided an estimate of visual acuity.  For the optomotor response (Supplemental Figure 3), mice were placed on a platform in the form of a grid (5 cm diameter, 10 cm above the bottom of the drum) surrounded by a motorized drum (26 cm diameter) that could be revolved clockwise or anticlockwise at two revolutions per min which is the optimal velocity for evoking an optokinetic response in the mouse [2, 3]. After 10 min of adaptation in the dark, vertical black and white stripes of a defined spatial frequency were presented to the animal. These stripes were rotated alternating between clockwise and anticlockwise, for 2 min in each direction with an interval of 30 s between the two rotations. Various spatial frequencies subtending 0.03, 0.09, 0.15, and 0.26 cycles/degree (cpd) were tested individually on different days in a random sequence. Animals were videotaped with a digital video camera (DCR-TRV24E, Sony, Tokyo, Japan) for subsequent scoring of head tracking movements. Tests were initially performed in scotopic conditions, using the night shot position of the camera. For photopic measurements, animals were subjected to 90,000 lux for 5 min to allow them to adapt to the light. Head movements were scored only if the angular speed of the head corresponded to that of the drum rotation. If the spatial frequency of the black and white stripes was increased, a threshold was reached beyond which no tracking movements of the head were detected. The visual spatial resolution (or visual acuity) of the animal was estimated to be greater than or equal to this threshold, but below the next spatial frequency tested. For the experiments described here, rotation speed of the virtual drum was kept constant at 12 degrees/second.

***Recording flash-visual evoked potential***

Mice were deeply anesthetized with 10% chloral hydrate (0.075ml/10g) by intraperitoneal injection. Temperature during surgery was maintained at 37°C using a heating pad. Flash-visual evoked potential (VEP) responses were recorded simultaneously using platinum needle electrodes. The VEP active electrode was placed in the scalp along the midline of the visual cortex. Platinum needles placed in the lower jaw and forelimb served as reference and ground leads, respectively. VEP recording was performed on the same eye (randomly selected). Visual stimuli were elicited by flashes of white light recorded on a dark background and photic stimulation was delivered at a frequency of 1.1 Hz. Stimulus intensity was 3.0 cd·s/m^2^, the flash duration was 5 ms and the interstimulus interval was 909 ms. Signals were amplified (×20K), band pass filtered (1–100 Hz), and digitized with 16 bits A/D conversion using a GT-2008V electrophysiology system (GOTEC LLC, Chongqing, China). At the intensity tested, 20 to 64 successive responses were averaged to obtain a mean VEP response. The standard flash VEP elicited a reproducible waveform characterized by negative (N) and positive (P) deflections. The amplitude and latency of the main positive components of flash-VEPs P2 were measured.

***Intravitreal injections***

To evaluate the effect of FGF2 on cellular function, an FGF2 neutralizing antibody (Cat#: 05-117, Millipore, Billerica, Massachusetts, USA) was used. For this purpose, at 3 months after BM reconstitution, I/R injury was induced in chimeric mice as described in the Materials and Methods section. The vitreous cavity of mouse eyes (approximately halfway between the zonule fibers and the apex of the cornea) was injected with 2 μl of the FGF2 neutralizing antibody (2μg/2 μl) or 2 μL of control vehicle after the onset of I/R injury. Western blots for detection of Bcl-1, Bax, phospho-Akt/total Akt were carried out at 3 days post I/R injury and the FGF2 neutralizing antibody injection. Retrograde labeling of retinal ganglionic cells was carried out at 7 days post I/R injury and the FGF2 neutralizing antibody injection.

***Retrograde labeling of retinal ganglionic cells and quantification***

Retrograde labeling of retinal ganglionic cells (RGCs) was achieved 3 days before I/R injury. Mice were deeply anesthetized and immobilized in a small stereotactic instrument with 10% chloral hydrate (0.075ml/10g) by intraperitoneal injection. The skull was exposed and bregma identified. A hole was drilled above the superior colliculus of each hemisphere. Using a Hamilton injector, 1μl Hydroxystilbamidine (4% in PBS, equivalent to FluoroGold; Abcam, Cambridge, MA, USA) was injected into both superior colliculi at a depth of 1 mm from the bony surface of the brain. The mice were sacrificed at 3 and 7 days post retinal injury. FluoroGold-positive RGCs were identified with a fluorescent microscope (Nikon, Tokyo, Japan) after preparation for a retinal flat mount. The average density of the viable RGCs (gold dots) was counted using Image J software (NIH, Bethesda, Maryland, USA).

***Organotypical retinal explant culture***

Primary organotypical retinal explants from aged C57BL/6 mice (20-22 months) dissected free from retinal pigmented epithelium and neural retina were then flat-mounted with the photoreceptor side down on polytetra-fluoroethylene membranes (EMD Millipore) on an air-fluid interface overlying serum-free culture media with penicillin and streptomycin (IMDM, Cat#: 12200-036, Invitrogen) in normoxic and hypoxic (0.1% O_2_) conditions. For co-culture experiments, BMSca-1^+^ and Sca-1^-^ cells from C57BL/6 mice 2-3 months of age were isolated using immunomagnetic activated cell sorting as described in the Materials and Methods section. The fresh sorted young BM Sca-1^+^ and Sca-1^-^ cells were washed with PBS before being suspended at a concentration of 5×10^5^ cells/ml in retinal explant culture media. A 30 μl droplet of cell suspension was placed on the RGC surface of each retinal explant. For treatment with inhibitors of FGF2, an FGF2 neutralizing antibody was added to the culture media at 20 ng/ml. After washing off the BM cells, the retinal explants were either fixed with 4% PFA or extracted for total protein at 48 hours after co-culture with BM Sca-1^+^ and Sca-1^-^ cells and the FGF2 neutralizing antibody under either normoxia or hypoxia conditions.

***TUNEL assay***

Terminal deoxynucleotidyl transferase dUTP nick end labelling (TUNEL) was carried out according to the manufacturer’s instructions (Roche, Laval, QC, Canada). The TUNEL Kit was used to label DNA strand breaks by terminal deoxynucleotidyl transferase (TdT), which catalyses polymerization of labelled nucleotides to free 3’-OH DNA ends in a template-independent manner (TUNEL reaction). In brief, the retinal explants were fixed with a freshly prepared fixation solution (4% PFA in PBS, pH 7.4) for 20 min at room temperature. After incubation with freshly prepared permeabilization solution (0.2% Triton X-100, 0.1% sodium citrate) for 15 min, the retinal explants were labelled using the TUNEL reaction mixture in a humidified atmosphere for 60 min at 37°C in the dark. The retinal explants were subsequently rinsed 3 times with PBS to stop the reaction. The nuclei were then stained with DAPI for 5 min at room temperature (Cat#: D9542, 1;2000, Sigma, St. Louis, MO, USA). The number of TUNEL^+^ cells in five randomly selected high-power fields per slide was determined and averaged with a Nikon Eclipase Ti fluorescent microscope. The number of animals used for each quantification is indicated in the figure legends.

***Enzyme-linked Immunosorbent Assay (ELISA)***

To determine the protein level of FGF2, IGF-1, CNTF, NGF, FGF1, SCF and NDNF, the culture medium and the lysate from 5×10^5^ cells per sample of the Sca-1^-^ and Sca-1^+^ cells were analyzed. The level of each growth factor was determined using ELISA following the manufacturer’s instructions (Cat#: MFB00, R&D Systems, Minneapolis, USA; IGF-1, Cat# ab100695, FGF1, Cat# ab223587, SCF, ab197750, Abcam, Cambridge, MA, USA; CNTF, Cat# OKEH04032, NGF, Cat# OKBB00230, NDNF, Cat# OKEH01931, Aviva Systems Biology, San Diego, USA) and normalized as pg/mg total protein.

***RNA extraction and RT-qPCR***

Total RNA was isolated from primary retinas according to the manufacturer’s recommendations using Trizol reagent (Sigma, St. Louis, Missouri, USA). Two mouse retinas were harvested as one sample for total RNA extraction. Reverse transcription was performed using SuperScript III (Invitrogen, Waltham, MA, USA) and 0.5 μg of total RNA served as the template for each reaction. Quantitative analysis was performed by qPCR using a SensiFAST SYBR No-ROX Mix Kit (Bioline, London, UK) following the standard protocol. The sequences of the mouse primers used are shown in Supplemental Table 1.

***Immunofluorescent staining and confocal microscopy***

The mice were sacrificed at 3 months post chimeras to identify the homed young BM cells in the retinas and mice were sacrificed at 3 and 7 days post retinal injury in chimeric mice to evaluate cell differentiation and visual function. The eye bulbs of mice were enucleated and fixed in 4% PFA overnight and then exposed to 20% sucrose for 24 hours at 4℃. The cultured retinal explant tissue was also fixed in 4% PFA at 4℃ overnight and then transferred to 20% sucrose for 24h at 4℃. All samples were then embedded in frozen optimum cutting temperature (OCT, Tissue-Tek, Somagen, Edmonton, Alberta, Canada) medium and cryosectioned at a thickness of 6μm using a cryotome. The sections were dried, followed by fixation with 2% PFA for 10min, and then permeabilization with 0.2% TritonX-100 for 5 min. After blocking with 1% bovine serum albumin for 30min at RT, the sections were incubated for 2h with the following primary antibodies: Alexa488 conjugated anti-GFP (Cat#: A21311, 1:400, Invitrogen, Carlsbad, California, USA) or Goat anti-GFP (Cat#: AB6673, Abcam, Cambridge, MA, USA), anti-Sca-1 (Cat#: AF1226, 1:100, R&D system, Minneapolis, MN,USA), Rat anti-F4/80 (Cat#: AB6640, 1:200, Abcam), Rabbit anti-GFAP (Cat#:Z0334, 1;200, DAKO, Santa Clara, CA, USA), Mouse anti-NeuN (Cat#: MAB377, 1:100, Millipore, Billerica, Massachusetts, USA). After washing, slides were then incubated with their corresponding Alexa 488 or 568 or 647 conjugated secondary antibodies (Invitrogen). The nuclei were stained with DAPI as described above. The number of positive cells in five randomly selected high-power fields per section was quantified using a Nikon Eclipase Ti fluorescent microscope. Co-localization of fluorescent signals was captured and visualized using an Olympus Fluoview 2000 laser scanning confocal microscope.

***Protein isolation and Western blotting***

Retina tissues were lysed in extraction solution (20mM Tris [pH 7.4], 150mM NaCl, 1mM EDTA, 1mM EGTA, 1% Triton, 2.5mM Na pyrophosphate, 1mM β-glycerolphosphate, 1mM Na_3_VO_4_, 1μg/ml of leupeptin, 1μg/ml of pepstatin, and 1mM phenylmethylsulfonyl fluoride) for 1h on ice. Lysates were separated by 10% sodium dodecyl sulfate polyacrylamide gel electrophoresis and transferred onto polyvinyl difluoride membranes. Membranes were blocked with a 5% skim milk solution and incubated with the following antibodies: p-ser473-Akt (Cat#: 9271, 1:1000, Cell Signaling, Danvers, MA, USA), total Akt (Cat#: 9272, 1:1000, Cell Signaling), Bcl2 (Cat#: B9804, 1:1000, Sigma, St. Louis, Missouri, USA), Bax (SC-7382, 1:500, Santa Cruz, Dallas, TX, USA), FGF2 (Cat#: 05-118, 1:500, Millipore), and GAPDH (Cat#: MAB374, 1:10000, Millipore). The blots were visualized with HRP-conjugated secondary antibodies and enhanced chemiluminescence. For quantification, densitometry of the target bands was divided by the corresponding densitometry of the GAPDH band using AlphaImager 2200 software (ProteinSimple, San Jose, CA, USA).

**References**

1. **Thiels E, Hoffman EK, Gorin MB**. A reliable behavioral assay for the assessment of sustained photophobia in mice. *Curr. Eye Res.* 2008; 33; 483–91.

2. **Mangini NJ, Vanable JW, Williams MA, et al.** The optokinetic nystagmus and ocular pigmentation of hypopigmented mouse mutants. *J. Comp. Neurol.* 1985; 241; 191–209.

3. **Mitchiner JC, Pinto LH, Vanable JW**. Visually evoked eye movements in the mouse (Mus musculus). *Vision Res.* 1976; 16; 1169–71.


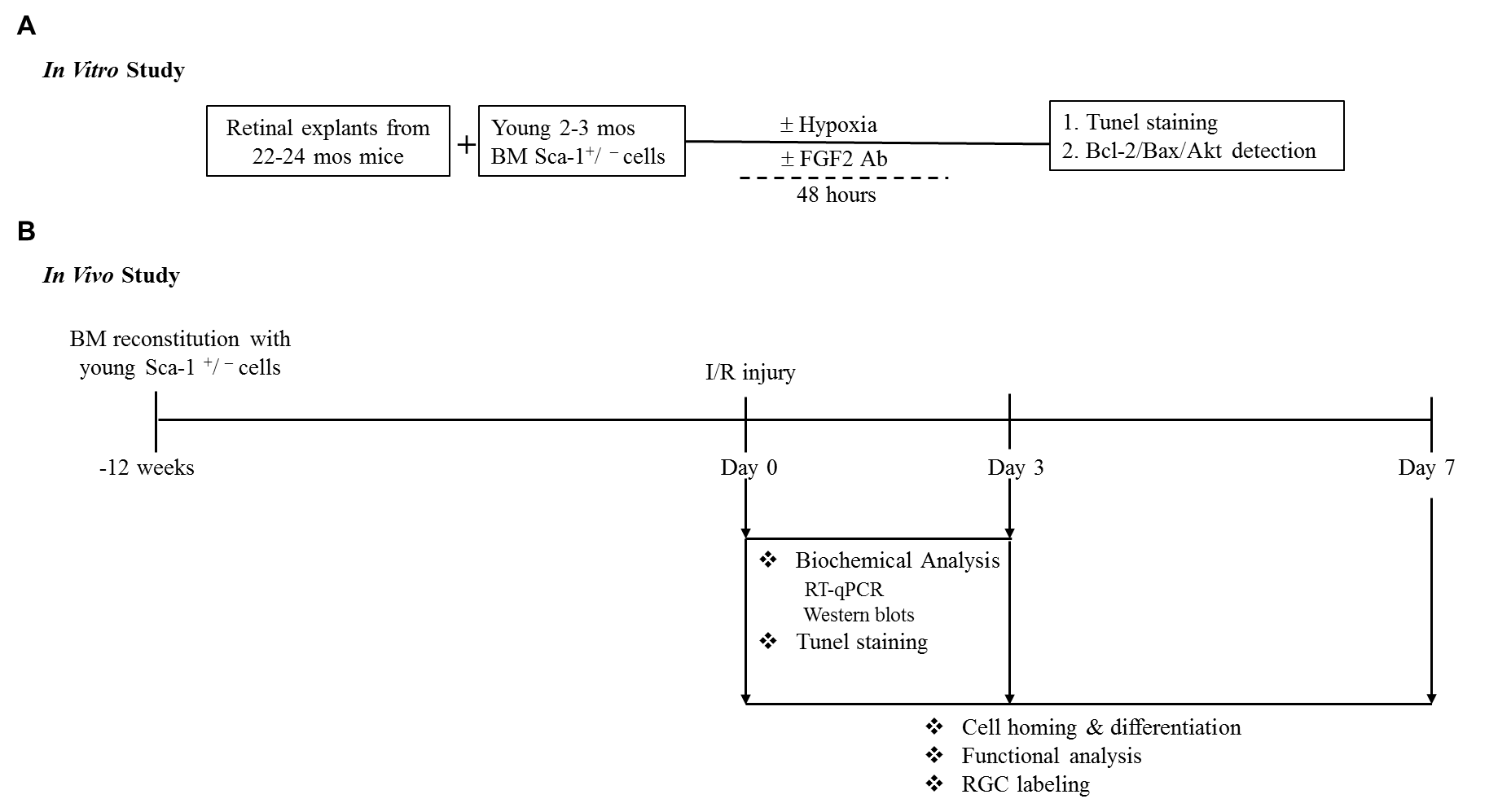


**Supplemental Figure 1.** Experimental timeline. **(A)** Schematic layout with procedures conducted in the *in vitro* study. **(B)** Schematic timetable with procedures conducted in the *in vivo s*tudy. BM: bone marrow; I/R: ischemia-reperfusion; FGF2 Ab: FGF2 neutralizing antibody.

**
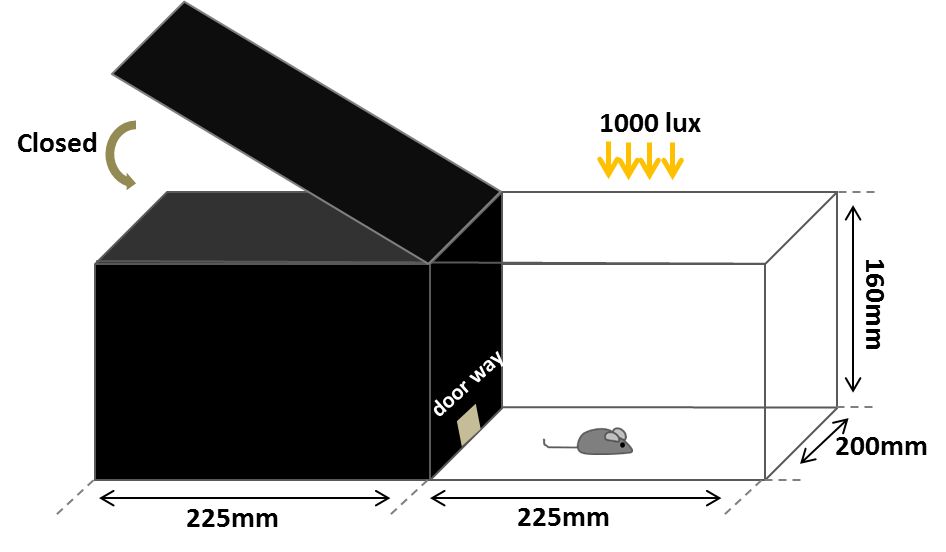
**

**Supplemental Figure 2.** Schematic diagram of the Light/Dark Box Exploration apparatus.


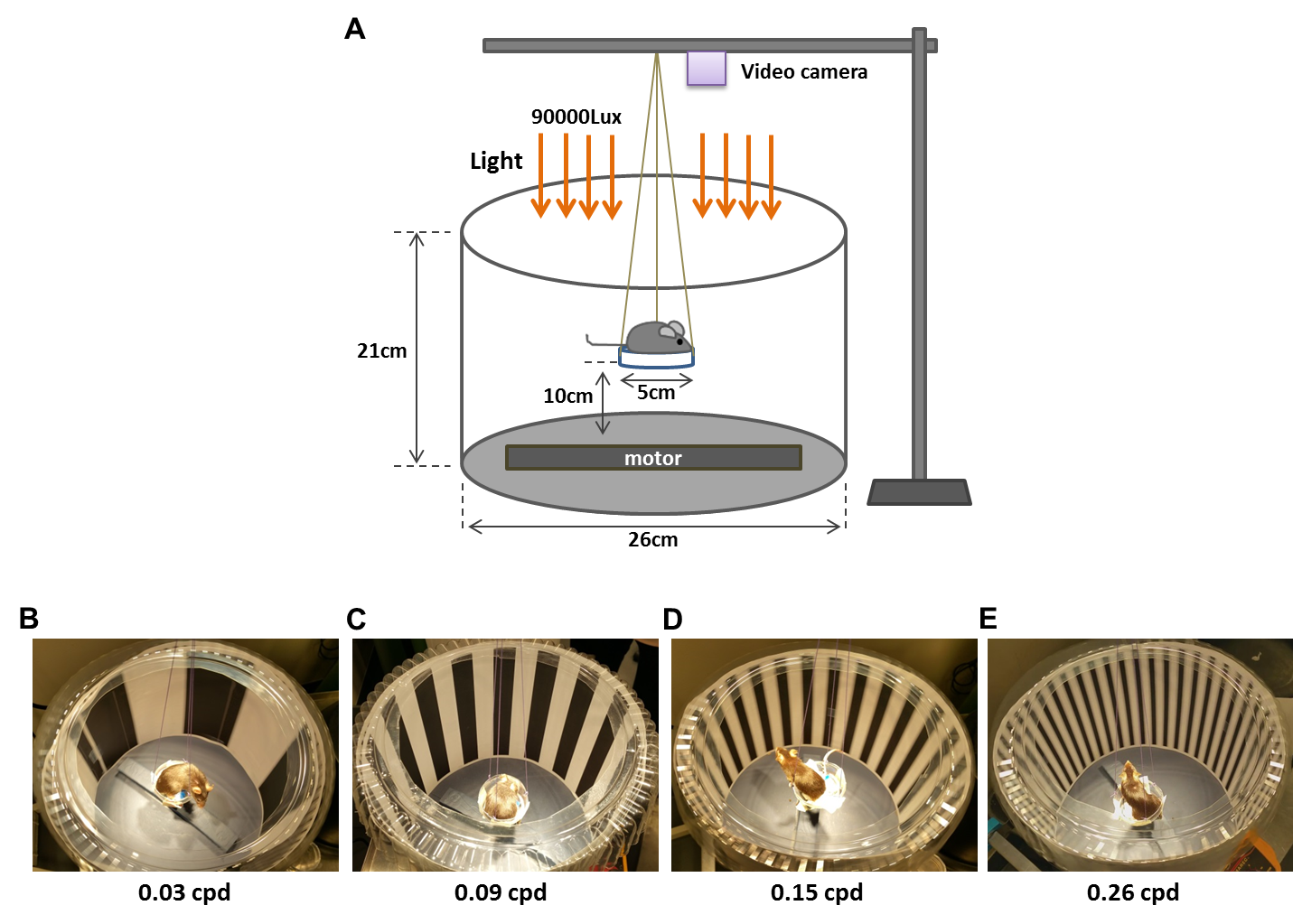


**Supplemental Figure 3.** Schematic diagram of the visual tracking drum for optomotor response testing. **(A)** Schematic diagram of the visual tracking drum. The mouse is placed on the pallet (5.0 cm diameter) which was raised 10 cm above the floor of the drum. The light (90,000 lux) was placed above the mouth of the drum and was operated via a light control and a video camera was held for tracking. **(B-E)** Spatial frequencies were tested at 0.03, 0.09, 0.15, and 0.26 cpd (cycles/degree).

**Supplemental Table 1. Sequences of mouse primers used in RT-qPCR.**

Reverse Transcription qPCR primers

| Gene | Primer sequences |
| --- | --- |
| FGF2（Forward） | 5’- TCTACTGCAAGAACGGCG -3’ |
| FGF2（Reverse） | 5’- CTCCCTTGATAGACACAACTCC-3’ |
| IGF-1（Forward） | 5’- TGCCTCTGTGACTTCTTG -3’ |
| IGF-1（Reverse） | 5’- GGCTCCTCCTACATTCTG -3’ |
| CNTF（Forward） | 5’- CGGTTGACTCAGTGGATGGT -3’ |
| CNTF（Reverse） | 5’- TGACACGGAGGTCATGGATA -3’ |
| NGF（Forward） | 5’- GCAGTGAGGTGCATAGCGTA -3’ |
| NGF（Reverse） | 5’- CACTGAGAACTCCCCCATGT -3’ |
| FGF1（Forward） | 5’-GCTGAAGGGGAGATCACAAC -3’ |
| FGF1（Reverse） | 5’-ACAGCTCCCGTTCTTCTTGA -3’ |
| SCF（Forward） | 5’- GAAGAGGCCAGAAACTAGAT -3’ |
| SCF（Reverse） | 5’- TGCGGCTTTCCTATTACTGAC -3’ |
| NDNF（Forward） | 5’- CTTCAAGGACCCAGAAGCTG -3’ |
| NDNF（Reverse） | 5’- GGGCTTCAGGTCAGAGACTG -3’ |
| GAPDH（Forward） | 5’- CTTTGTCAAGCTCATTTCCTGG -3’ |
| GAPDH（Reverse） | 5’- TCTTGCTCAGTGTCCTTGC -3’ |

Primers used for Reverse Transcription (RT)-qPCR of FGF2 (Fibroblast Growth Factor 2), IGF-1 (Insulin-like Growth Factor 1), CNTF (Ciliary Neurotrophic Factor), NGF (Nerve Growth Factor), FGF1, SCF (Stem Cell Factor), NDNF (Neuron-Derived Neurotrophic Factor), and GAPDH quantification.
